# Environmental and Dispersal-Related Drivers of Color Morph Distribution in *Triatoma infestans* (Klug, 1834) (Hemiptera, Reduviidae)

**DOI:** 10.3390/insects16111103

**Published:** 2025-10-29

**Authors:** Erika V. Díaz, Federico G. Fiad, Gisel V. Gigena, Ana G. López, Romina V. Piccinali, Ana Laura Carbajal-de-la-Fuente, Claudia S. Rodríguez, Julieta Nattero

**Affiliations:** 1 Cátedra Morfología Animal, Facultad de Ciencias Exactas, Físicas y Naturales Universidad Nacional de Córdoba, Avenida Vélez Sarsfield 299, Córdoba X5000, Argentina; erika.diaz@mi.unc.edu.ar (E.V.D.); federico.fiad@mi.unc.edu.ar (F.G.F.); gisel.gigena@mi.unc.edu.ar (G.V.G.); ana.lopez@unc.edu.ar (A.G.L.); 2 Instituto de Investigaciones Biológicas y Tecnológicas (IIBYT-CONICET), Córdoba X5000, Argentina; 3 Cátedra Introducción a la Biología, Facultad de Ciencias Exactas, Físicas y Naturales Universidad Nacional de Córdoba, Avenida Vélez Sarsfield 299, Córdoba X5000, Argentina; 4 Laboratorio de Eco-Epidemiología, Departamento de Ecología, Genética y Evolución, Facultad de Ciencias Exactas y Naturales, Universidad de Buenos Aires, Intendente Güiraldes 2160, Ciudad Autónoma de Buenos Aires B1428, Argentina; rpicci@ege.fcen.uba.ar; 5 Instituto de Ecología, Genética y Evolución de Buenos Aires (IEGEBA), CONICET-Universidad de Buenos Aires, Ciudad Autónoma de Buenos Aires B1428, Argentina; 6 Centro Nacional de Diagnóstico e Investigación en Endemo-Epidemias (CeNDIE), Administración Nacional de Laboratorios e Institutos de Salud “Dr. Carlos Malbrán” (ANLIS), Avenida Paseo Colón 568, Ciudad Autónoma de Buenos Aires B1063, Argentina; analaura.carbajal@gmail.com; 7 Consejo Nacional de Investigaciones Científicas y Técnicas (CONICET), Ciudad Autónoma de Buenos Aires C1040, Argentina; 8 Departamento de Biodiversidad y Biología Experimental, Facultad de Ciencias Exactas y Naturales, Universidad de Buenos Aires, Intendente Güiraldes 2160, Ciudad Autónoma de Buenos Aires B1428, Argentina

**Keywords:** climatic variables flight estimators, flight-related traits, chromatic morphotypes, vegetation cover, wing loading

## Abstract

Understanding the dispersal capacity of *Triatoma infestans*, the main vector of Chagas disease in South America, is vital for vector control and managing recolonization after insecticide use. This study compares the seasonal frequency of melanic and non-melanic *T. infestans* morphs in Northwestern Córdoba Province, Argentina, and examines their association with environmental variables, morphometric traits, nutritional status, and flight capacity. Insects were collected at the beginning and end of the warm season. Dorsal coloration, morphometric traits, nutritional status, flight-related indices, climatic variables, and vegetation cover were recorded. Chromatic morph frequencies were analyzed using chi-square tests. Biological predictors were identified through multi-model inference, and environmental associations explored with Canonical Correspondence Analysis. Melanic individuals decreased from early to late warm season, especially males. Wing loading correlated strongly with morphotype, being higher in non-melanic forms. Pronotum size were also a significant predictor. Nutritional status had no clear effect. Cattle pasture cover and rainfall influenced morph frequency, mainly in males. These results reveal a complex interaction between phenotypic and environmental factors shaping color morph variation, highlighting the importance of understanding these dynamics to optimize vector surveillance and control in areas prone to reinfestation.

## Simple Summary

*Triatoma infestans* is a blood-feeding insect and the main vector responsible for transmitting the parasite that causes Chagas disease in southern South America. This species shows different color forms—some individuals are darker (melanic), while others are lighter (non-melanic). In this study, we examined the presence of these two forms in a rural region of Argentina, comparing how common they were at the beginning and end of the warm season. We also looked at whether their physical characteristics, flight potential, body condition, and the surrounding environment affected which form was more frequent. We found that dark individuals were more common early in the season but decreased over time, especially in males. Lighter insects tended to have body traits that may help them fly better. Environmental factors such as pasture coverage and rainfall also appeared to influence which form was present. These results suggest that both biological and environmental factors play a role in shaping the physical appearance of these insects. Understanding this variation can help improve monitoring and control efforts, especially in areas where insects may return after insecticide spraying.

## 1. Introduction

*Triatoma infestans* (Klug, 1834), one of the 158 recognized species of triatomines (Hemiptera: Reduviidae: Triatominae) [1], is the main vector of the etiological agent of Chagas disease in southern South America [2,3]. This insect is widely distributed across a latitudinal gradient [4,5], and is successfully adapted to thrive in human dwellings and other human-made or modified structures used by domestic animals (peridomiciles) [6,7]. The Southern Cone Initiative to Control Chagas Disease (INCOSUR) succeeded in reducing the distribution area of *T. infestans* based on vector control, health education and house improvement programs [8]. Nevertheless, in the arid zones of the Gran Chaco and Monte Desert in Argentina, Paraguay, and Bolivia, reinfestations of human dwellings continue to occur in several provinces or departments [9,10,11]. Additionally, residual foci of *T. infestans* have been reported in Rio Grande do Sul, Brazil [12]. Across the Gran Chaco region, reinfestation events of *T. infestans* typically involve the active dispersal of insects from sylvatic habitats, pyrethroid-resistant domestic populations, or peridomestic populations, which serve as the main foci of reinfestation following insecticide application [13,14,15,16,17,18]. In this context, the dispersal capacity of these insects is a crucial factor influencing the long-term success of vector control interventions, as dispersing individuals can invade or recolonize treated ecotopes, which is the most important mechanism of home invasion [6]. Dispersal involves the movement of individuals or populations from their birth or breeding site to a new location [19], with potential consequences for gene flow across space [20]. In triatomines, flight is recognized as the main means of active dispersal by which adult individuals move within the environment. They also exhibit sexual dimorphism in this process due to physiological and behavioral differences [21,22]. The nutritional and reproductive status of triatomines, population density, and environmental characteristics are among the main drivers of this dispersal process [23,24,25].

Environmental factors can drive adaptations of flight-related traits to improve dispersal efficiency in insects [26]. Head and wing size and shape are key determinants of dispersal and influence flight performance, orientation, and energetic efficiency [27,28,29,30]. Additionally, flight performance can be studied through estimators such as aspect ratio and wing loading [31]. In triatomines, climatic conditions and vegetation cover in the ecotopes act as modulating factors during development, influencing overall body size as well as the structure and shape of flight-related characters [28,32,33,34,35,36,37,38,39]. Recently, significant correlations between temperature, precipitation, and latitude with forewing size and shape have been reported for *T. infestans* [40]. Furthermore, changes in the shape of the head and compound eyes are also associated with triatomine flight performance [37,38,41].

In nature, organisms of the same species commonly display phenotypic differences that determine external signals of their interaction with the environment, through traits barely perceptible to the naked eye, such as wing, head, and body shape, or visible traits, such as coloration. Color variations, such as melanism, manifest themselves in variants with completely dark or predominantly dark shades [42]. Melanism is very common in insects, where melanic or dark forms exhibit an unusually high concentration of melanin pigments that act as important structural and protective components of the cuticle [43]. Individuals with dark color patterns would be at an advantage under low ambient temperature and high UV radiation conditions and should be less tolerant to desiccation [44,45]. The first records of melanistic coloration patterns for *T. infestans* were reported from wild foci in the Bolivian Chaco [46,47]. In Argentina, various authors have reported melanism in a wild population associated with parrot nests [48] in domiciles in the Chaco Province (Department of General. Güemes) [49], and in peridomiciles of the Córdoba Province (Department of Cruz del Eje) [50]. Furthermore, melanic populations of *T. infestans* from Misiones in northwestern Argentina were long considered a valid taxon *T. melanosoma* Martínez, Olmedo & Carcavallo, 1987 [51]. Since 1999, this species has been considered synonymous with *T. infestans* [52]. Melanic coloration could be an adaptive character or be linked to other adaptive traits (i.e., pleiotropic effects) [42]. For *T. infestans*, morphometric variations found between melanic and non-melanic groups were suggested to be due to different ecological adaptations presented by each chromatic morph [50].

The northwestern region of the Córdoba Province, located south of the Argentine Gran Chaco, presents a heterogeneous scenario of *Trypanosoma cruzi* (Chagas, 1909) (Kinetoplastida, Trypanosomatidae) transmission, etiological agent of Chagas disease. This is related to differences in vector control interventions, changes in land use, and socioeconomic factors in recent decades [53,54]. This region has a historical occurrence of *T. infestans* and records of wild species invading domestic settings [55]. In particular, the Cruz del Eje Department, located in the west of the province, has numerous areas with recurrent infestations of houses by *T. infestans* and other triatomine species [16,54]. For this area, there is a single recorded instance of a wild collection of *T. infestans* approximately 115 m from a dwelling [55].

Although many studies have investigated morphometric variation in triatomines and highlighted its value as an indicator of microevolutionary dynamics, no studies have explored the relationship between dispersal-related and environmental factors as determinant of the presence of *T. infestans*, especially in association with the presence of chromatic variants (melanic and non-melanic forms) within a reinfestation context. Dispersal events of *T. infestans* between sylvatic and domestic environments, followed by colonization, may occur more frequently than previously assumed [48,56]. Recognizing melanic forms in both domestic and peridomestic settings—and associating them with dispersal potential and ecological factors—is therefore relevant to understanding their possible epidemiological importance. Moreover, assessing the potential role of these morphs in the reinfestation of houses after chemical control interventions is crucial for enhancing vector management strategies. In this study, we aim to compare the frequency of melanic and non-melanic forms of *T. infestans* between seasons in a defined rural area of northwestern Córdoba Province, Argentina; to explore how these frequencies relate to environmental variables (e.g., vegetation cover, mean temperature and precipitation) and morphometric traits (e.g., forewing size, head and pronotum measurements), nutritional status and flight estimators (wing loading and aspect ratio) and to identify the most relevant predictors of each morph presence. We hypothesize that the frequency of melanic and non-melanic *T. infestans* forms varies between seasons, and is associated with environmental and phenotypic variables. Specifically, we expect melanic forms to be more frequent in cooler or drier conditions and to differ in morphometric and flight-related traits compared to non-melanic individuals. This integrative approach seeks to deepen our understanding of *T. infestans* phenotypic variation and its implications for vector ecology and control.

## 2. Materials and Methods

### 2.1. Study Area

**Figure 1:**
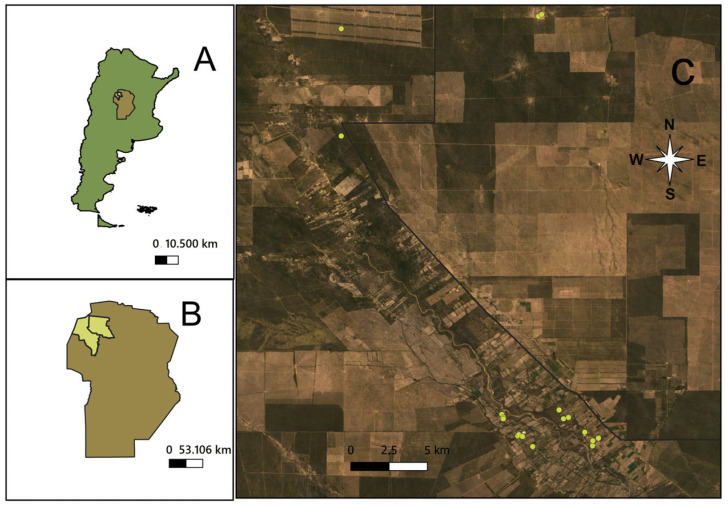
Maps of the study area. (**A**). Location of Córdoba Province (brown) within Argentina. (**B**). Location of Cruz del Eje and Ischilín Departments (light green) within Córdoba Province. (**C**). Detailed view of the study area showing the individual houses included in the study. The compass rose shows the cardinal directions.

Insects were collected from domestic and peridomestic sites in a rural area (approximately 20 × 30 km) located in the departments of Cruz del Eje and Ischilín, in the northeastern region of Córdoba Province, Argentina, situated at the southern edge of the Gran Chaco region (Figure 1).

The study was carried out between 2012 and 2013 in coordination with the surveillance and vector control activities carried out by the Provincial Chagas Program of Córdoba Province. Collections were conducted in December (beginning of the warm season) and April (end of the warm season). In December 2012, the rural villages of Palo Parado, Villa Luján, Guanaco Muerto and Las Casillas, all within the department of Cruz del Eje, were visited (Figure 1). In April 2013, collections were conducted in La Concepción (Ischilín Department) and El Simbolar (Cruz del Eje Department). The study area corresponds to the phytogeographic Chaco region. The climate is semi-arid, with most of the annual precipitation—ranging from 400 to 700 mm—falling during the summer months. This region is part of the Arid Chaco, historically known as endemic for Chagas disease. Summers are typically very hot, with temperatures often exceeding 40 °C, while winter temperatures can fall below 5 °C [57]. In this area, a 5-year historical record up to the sampling date shows that, in December, the mean air temperature was 25.31 °C, with a minimum of 18.24 °C and a maximum of 32.38 °C. Rainfall averaged 53.77 mm, and the relative humidity was 26.63%. For April, the mean air temperature was 19.83 °C, with a minimum of 13.05 °C and a maximum of 26.61 °C. Rainfall averaged 21.50 mm, and the relative humidity was 56.88%. The terrain consists of gently rolling plains with saline-alkaline soils and diverse native vegetation, including forests of *Aspidosperma quebracho-blanco*, *Prosopis* spp., *Celtis* spp., and *Geoffroea decorticans*, as well as shrublands dominated by *Mimozyganthus carinatus* and *Larrea divaricata* [58]. The last community-wide insecticide spraying campaign using pyrethroids in this region was conducted by the National Vector Control Program three years before this study. Fieldwork was conducted in domiciliary units, which included both the interior of dwellings and the corresponding peridomestic areas. The latter typically consisted of attached structures such as chicken coops, goat or pig corrals, and, less frequently, rabbit hutches or storage facilities. Technical staff from the Provincial Chagas Program of Córdoba inspected a total of 91 domiciliary units (62 in December 2012 and 29 in April 2013) for the presence of *T. infestans*. Following inspection, all infested dwellings were treated with beta-cypermethrin flowable, at a 5% concentration (Sipertrin, Chemotécnica, Argentina) [59].

### 2.2. Insects

In December 2012, 62 houses were inspected, and *T. infestans* was found in 33 of them (53%). Adult specimens were present in 16 of the infested houses. In April 2013, a total of 29 houses were inspected, and 11 of them had adults present. Triatomine searches were conducted in both intra- and peridomestic structures using the person-hour technique [60]. One person inspected each site of the domiciliary unit for 20 min to search for triatomines. All collected specimens (103 adults and 217 nymphs in December and 66 adults and 75 nymphs in April) were transported to the laboratory under refrigerated conditions, where species, sex and nymphal stage were determined for each individual following Lent & Wygodzinsky [60]. All adult *T. infestans* were examined for the presence of *T. cruzi* in feces using conventional light microscopy; none tested positive for the parasite. The weight (W) of adults was recorded using an electronic balance (precision ± 0.001 g; Mettler, Denver, CO, USA). Digital photographs of all adults were taken in dorsal view, on a white background, with a reference scale, under standardized lighting conditions and with the camera in a fixed position. Subsequently, insects were dissected, and the forewings, head, and pronotum were removed. These structures were photographed using a Moticam 2 digital camera attached to a stereomicroscope (Stemi 2000-C; Zeiss, Oberkochen, Germany), at 6× magnification with a reference scale. In total, 167 individuals were analyzed (50 females and 55 males in December 2012, and 28 females and 33 males in April 2013).

### 2.3. Colorimetric Analysis

The dorsal coloration of *T. infestans* adults was quantified from digital images of the right forewing and right connexivum of each individual, following the methodology described by Nattero et al. [50]. Colorimetric quantification was performed on known and homologous portions of both structures. A 15 × 36 mm section of the forewing, which includes both the membranous and stiff portions of the forewing, and a 1.5 × 18 mm section of the connexivum were extracted. The forewing was selected due to the presence of a yellowish spot in its upper third, visible only in lighter-colored individuals. Colorimetric analysis was carried out using Image Color Summarizer v 0.76 “https://mk.bcgsc.ca/colorsummarizer/ (accessed on 24 October 2025)”). For each individual, the average values of the red, green, and blue (RGB) components were obtained for both the forewing and the connexivum, resulting in six colorimetric variables per specimen. To identify clustering patterns in color characteristics, a K-means clustering analysis was applied. The six variables derived from the RGB threshold analysis were used in K-means clustering for each sampling season. The optimal number of chromatic groups was determined using the elbow method [61].

### 2.4. Nutritional Status

The nutritional status of the triatomines was estimated using the ratio between body weight (W, mg) and total body length (L, mm), expressed as W/L (mg/mm). This index is commonly used as an indicator of nutritional condition in triatomines [21,62,63]. To properly account for the difference in units between weight and length, the nutritional index was corrected by dividing weight by length cubed (W/L^3^), resulting in units of mg/mm^3^.

### 2.5. Linear Morphometric Measurements

**Figure 2:**
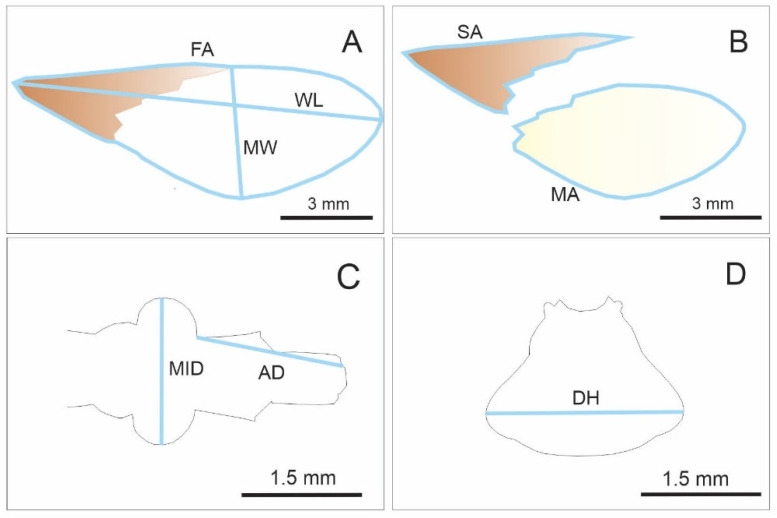
Measurements taken on the right forewing (**A**), stiff (brown) and membranous (light brown) portions of the forewing (**B**), head (**C**), and pronotum (**D**) in color morphotype adults of *Triatoma infestans* from a well-defined rural area in Córdoba, Argentina. All measurements are shown in light blue. Abbreviations: WL, forewing length; WW, maximum forewing width; FA, forewing area; SA, stiff portion of the forewing area; MA, membranous portion of the forewing area; MID, maximum interocular distance; AD, anterocular distance; DH, distance between the humeri.

Linear measurements were taken for total body length and the distance between the humeri (DH) (Figure 2D). Total body length was measured as the linear distance from the clypeus to the tip of the last abdominal segment. Additionally, linear measurements of the head were recorded, including the anteocular distance (AD) and the maximum interocular distance (MID) (Figure 2C). AD was measured from the anterior base of the eye to the distal end of the head, while MID corresponded to the maximum distance between the outer edges of both eyes (Figure 2C). For the right forewings, length (WL) and maximum width (MW) were measured (Figure 2A), along with total forewing area (FA) and the portions corresponding to the membranous (MA) and stiff areas (SA) (Figure 2B). All measurements were taken from digital photographs using ImageJ software, version 1.52 “https://imagej.net/ij/ (accessed on 24 October 2025)”.

### 2.6. Flight Performance Estimators

Two indicators of flight performance were calculated: wing loading (WL) and aspect ratio (AR). Wing loading was estimated following the methodology proposed by Martínez-Pérez et al. [31], as the ratio between the insect’s dry weight (mg) and the total forewing area (mm^2^), according to the formula: WL = dry weight/wing area. To calculate the aspect ratio (AR), the formula proposed by Betts and Wootton [64] was used, as applied to triatomines by Nattero et al. [35] and Verly et al. [41]: AR = 4 × (forewing length (mm)^2^/forewing area (mm^2^). Since the insects used in this study were weighed fresh, dry weight was estimated for both males and females based on a fresh-to-dry weight ratio. For this, 40 adult *T. infestans* individuals of each sex, with varying weights, were provided by UnOVE (CeNDIE, ANLIS/Malbrán, Santa María de Punilla, Córdoba, Argentina). The fresh weight of each specimen was recorded, followed by dry weight measurement after oven-drying at 25 °C in a Garmont oven. Based on these data, linear regression models were constructed to estimate dry weight for the individuals analyzed in this study, resulting in the following equations: for males: y = 0.29x + 0.01 and for females: y = 0.34x + 0.01, where x is the fresh weight and y is the estimated dry weight.

### 2.7. Climatic Variables and Vegetation Cover Estimation

Climatic data were extracted using Terra Climate (University of Idaho Data Store) [65] at a spatial resolution of 4.64 × 4.64 km, covering December (2008–2012) and April (2009–2013) of the five years preceding the sampling. Only data from these specific months were included, not the full year’s data. We limited our analysis to monthly mean temperature and monthly rainfall, variables that play a significant role in the distribution of Triatominae and are associated with insect flight dispersal [40,66,67]. To characterize vegetation cover, we used a 2013 land cover map from the MapBiomas Argentina “https://argentina.mapbiomas.org/en/ (accessed on 25 October 2025)” repository for the Cruz del Eje and Ischilín Departments. The maps have a spatial resolution of 30 m, allowing for detailed analysis of landscape dynamics over time. In order to study the relationship between the frequency of invasion of the melanic individuals to dwellings and vegetation cover characteristics, the coverage classes of the thematic map were grouped into four categories: Closed woody, Open woody, Cattle pastures, and Naked soil. Then, the localities were grouped into six square zones of 64 km^2^, and for each zone, the number of patches (NP) and the percentage of landscape (PLAND), two compositional landscape metrics, were extracted. The extraction of the metrics was carried out using the software FRAGSTATS 4 [68].

### 2.8. Data Analysis

A Principal Component Analysis (PCA) was performed to analyze the distribution of the chromatic groups identified through K-means clustering in the space defined by the first two principal components. Pearson’s X-squared test with Yates’ continuity correction was used to compare the frequency of melanic adults, females and males across samplings (beginning and end of the warm season) using the chisq.test function of R (version 4.4.1) executed in RStudio (RStudio, PBC, Boston, MA, USA) [69]. Goodness of fit tests were used to compare the frequency of melanic and non-melanic females and males within each sampling period using the same function in R. To evaluate the influence of the different studied variables—morphometric traits, nutritional status, and flight performance estimators—on the determination of chromatic morphotypes, a logistic regression with multi-model inference analysis was performed for each sex. Three separate multi-model inference analyses were conducted for each sex, one for each explanatory variable group: one for morphometric variables, one for nutritional status, and one for flight performance estimators. For each inference, model selection was based on the Akaike Information Criterion (AIC), and models with a ΔAIC < 3 were considered [70]. Akaike Information Criterion was corrected for small sample sizes (AICc), and model-averaged coefficients were calculated using full averaging. Before model fitting, collinearity among variables was assessed by evaluating the VIF of each variable; only those variables that exhibited a VIF < 5 were included as predictor variables for multi-model construction. All variables included in the analysis were standardized (mean = 0, SD = 1). The response variable, which modelled the chromatic morphotype of each individual, was modelled using a binomial distribution, considering two morphotypes, melanic and non-melanic, with a logit link function. The morphometric multimodel inference started from a full model; subsequently, a final averaged model was computed using this selection to estimate the parameter values. Model assumptions, including the appropriate distribution of the response variable, independence of observations, and absence of overdispersion, were verified for all models. All modelling procedures were carried out using R (version 4.4.1) executed in RStudio (RStudio, PBC, Boston, MA, USA). To assess the predictive performance of the model, the area under the curve (AUC) was used, as it is a reliable indicator of binary classification model quality. An AUC close to 1 indicates a model with strong predictive power. To examine the relationship between the frequency of color morphotypes and environmental variables (climatic and vegetation cover variables), Canonical Correspondence Analysis (CCA) was performed using the vegan package [71] in R (version 4.4.1) executed in RStudio (RStudio, PBC, Boston, MA, USA). The response matrix consisted of either the frequency of melanic females or males. In contrast, the explanatory matrix included the following environmental variables: the percentage of cover for closed woody vegetation, open woody vegetation, managed grassland and area without vegetation, precipitation and mean temperature. Total inertia (scaled Chi-square) was partitioned into constrained and unconstrained components. The significance of the global model, individual canonical axes, and each environmental variable was tested using 1000 permutations. Eigenvalues, the proportion of variance explained by each axis, and *p*-values from permutation tests were used to assess the explanatory power and statistical significance of each CCA model.

## 3. Results

### 3.1. Colorimetric Analysis, Seasonal and Sex-Specific Variation in the Frequency of Chromatic Morphotypes

**Figure 3:**
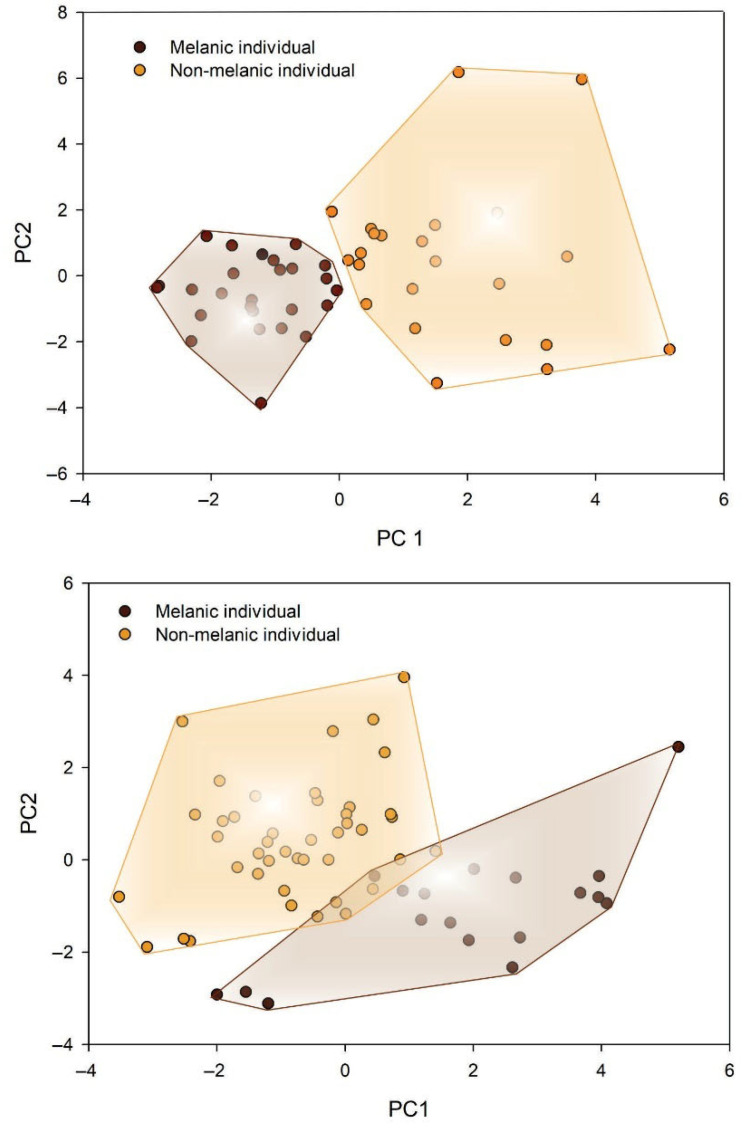
Biplots derived from the first two axes of principal components analyses showing the spatial distribution of the two clusters derived from a K-means clustering analysis (K = 2) for the beginning of the warm season sampling (**A**) and the end of the warm season sampling (**B**). For easy visualization, the lines connect the most external individuals of each chromatic group.

For both the beginning and end of the warm season samplings, the optimal number of clusters, as determined by the elbow method, was two. At the beginning of the warm season, the Principal component analysis (PCA) showing the distribution of individuals within the two clusters revealed relatively well-defined melanic and non-melanic groups, with the non-melanic group exhibiting greater variability than the melanic group (Figure 3A). At the end of the warm season, the PCA similarly indicated relatively well-defined melanic and non-melanic groups, with both groups displaying comparable levels of variability (Figure 3B).

Of the 105 adults collected at the beginning of the warm season, 43 (40.95%) were assigned to the melanic morphotype, while 18 (29.5%) of the 61 adults collected at the end of the warm season were melanic (X^2^ = 1.710, df = 1, *p* = 0.191) (Table 1). Melanic females did not show different frequencies between the beginning and end of the warm season (X^2^ = 1.311, df = 1, *p* = 0.252). However, melanic males were more frequent at the beginning than at the end of the warm season (X^2^ = 9.318, df = 1, *p*-value = 0.002) (Table 1). At the beginning of the warm season, the number of non-melanic females was significantly higher than the melanic (X^2^ = 8, df = 1, *p*-value = 0.005), while the number of non-melanic and melanic males did not show significant differences (X^2^ = 0.020, df = 1, *p* = 0.893). At the end of the warm season, this tendency was reversed, the number of melanic and non-melanic females did not show significant differences (X^2^ = 0.571, df = 1, *p*-value = 0.450), while the number of non-melanic males was significantly higher than the melanic (X^2^ = 13.364, df = 1, *p* < 0.0001).

### 3.2. Morphometric Variation Explains Chromatic Morphotypes in a Season- and Sex-Dependent Manner

**Figure 4:**
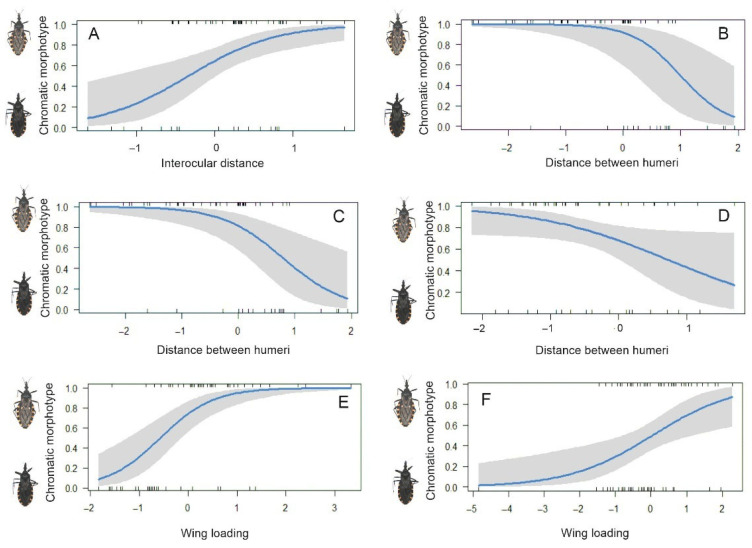
Predicted probability plots for the chromatic morphotypes of female and males of *Triatoma infestans* in relation to the significant predictor variables included in the multi-model inference analyses. Confidence intervals for each predictor are shown in grey. (**A**,**B**) males at the beginning of the warm-season (**A**) interocular distance, (**B**) distance between humeri). (**C**) females at the end of the warm-season sampling (distance between humeri). (**D**) males the end of the warm-season sampling (distance between humeri). (**E**) females (wing loading). (**F**) males (wing loading).

Results from the full models for females and males, which included all measured morphometric variables (forewing length, maximum forewing width, forewing area, stiff portion area, membranous portion area, maximum inter-eye distance, anterocular distance, and distance between the humeri) and the season as a fix factor, showed that the season had a Variance Inflation Factor (VIF) of 131.29 and 166.71 for females and males, respectively. Since the aim of this study is to analyze the effect of different morphometric variables within each sampling moment rather than to compare them directly across seasons, we ran separate Generalized Linear Models (GLMs) for females and males at each time point. For females at the beginning of the warm season, the multi-model inference approach identified the top five models with the lowest AICc values (Table S1). The top-ranked model had the greatest support among the candidate models (Akaike weight = 0.227); however, several competing models also showed non-negligible support (weights between 0.156 and 0.122), indicating moderate model selection uncertainty. Within the average model, interocular distance had a marginally significant positive effect (β = 0.98, Table 2) associated with a higher probability of being non-melanic. In contrast, anterocular distance, membranous portion area and distance between the humeri showed a negative effect, with a marginally significant effect for anterocular distance (Table 2). The odds ratios (ORs) supported these effects: interocular distance increased the odds of being non-melanic (OR = 3.314), while anterocular distance, membranous portion area and distance between the humeri reduced them (OR < 1) (Table 2). The average model showed good predictive performance, with an AUC of 0.787, indicating good discrimination between melanic and non-melanic individuals. For males at the beginning of the warm season, the top-ranked model had the greatest support among the candidate models (Akaike weight = 0.317); however, several competing models also showed non-negligible support (weights between 0.204 and 0.102), indicating moderate model selection uncertainty. The average model included interocular distance, forewing length, forewing width, stiff portion area, membranous portion area and distance between the humeri. Within this model, interocular distance, forewing length, forewing width and membranous portion area had a significant positive effect associated with a higher probability of being non-melanic. In contrast, the distance between the humeri showed a negative effect (Table 2). Both interocular distance and distance between humeri had significant effects (Figure 4A,B). The ORs supported these effects: interocular distance increased the odds of being non-melanic (OR = 2.57), while anterocular distance, membranous portion area and distance between the humeri reduced them (OR < 1) (Table 2). The average model showed good predictive performance, with an AUC of 0.907, indicating strong discrimination between melanic and non-melanic individuals.

For females, at the end of the warm season, the multi-model inference approach identified the top five models with the lowest AICc values (Table S2). While the top-ranked model had the highest Akaike weight (0.363), the presence of several other models with substantial weights (0.147–0.106) highlights moderate model selection uncertainty. The average model included: interocular distance, anterocular distance, forewing length and the distance between the humeri (Table 3). Specifically, distance between humeri showed a significant negative effect (β = −2.17) (Table 3, Figure 4C). The odds of being non-melanic are reduced with the distance between humeri (OR = 0.08) (Table 3). The average model demonstrated good predictive performance, with an AUC of 0.89, suggesting a high discrimination (89%) between melanic and non-melanic individuals. For males at the end of the warm season, although the top model had the highest weight (0.370), several alternatives with weights between 0.243 and 0.093 also received notable support, indicating some uncertainty in model selection. The average model distance between the humeri had a significant negative effect associated with a higher probability of being melanic (β = −1.98) (Table 3, Figure 4D). The ORs supported this effect, the distance between humeri increased the odds of being melanic (OR = 0.10) (Table 3). The average model showed good predictive performance, with an AUC of 0.908, indicating strong discrimination between melanic and non-melanic individuals.

### 3.3. Nutritional Status Fails to Predict Chromatic Morphotypes Across Seasons

Due to high collinearity for the fix factor sampling (VIF = 14.998 for females, 12.035 for males) and the inclusion of a single explanatory variable (nutritional index), models were run separately for each season without multi-model inference. For females, the model inference approaches indicated that the nutritional index did not show significant effects on the probability of being melanic or non-melanic from neither the beginning of the warm season (β = −0.144, *p* = 0.405) nor the end (β = 0.468, *p* = 0.185). For males the same tendency was observed, nutritional status did not show significant effects on the probability of being melanic or non-melanic from neither the beginning of the warm season (β = −0.192, *p* = 0.203) nor the end (β = 0.569, *p* = 0.218).

### 3.4. Wing Loading as a Significant Predictor of Chromatic Morphotype

For females, the multi-model inference approach identified the top three models with the lowest AICc values (Table S3). The top-ranked model had the lowest AICc and substantial support (weight = 0.587), although some degree of model selection uncertainty remained. Wing loading show a positive and significant effect on the chromatic morphotype (β = 1.83) associated with a higher probability of being non-melanic (Table 4, Figure 4E). Wing loading increased the odds of being non-melanic (OR = 6.35) (Table 4). The average model demonstrated very good discrimination ability, with an AUC of 0.85, indicating strong classification performance between melanic and non-melanic individuals. For males, the multi-model inference approach identified the top two models with the lowest AICc values (Table S3). The top-ranked model had the lowest AICc and substantial support (weight = 0.746), although some degree of model selection uncertainty remained. As for females, average model showed that wing loading show a positive and significant effect on the chromatic morphotype (β = 0.84) associated with a higher probability of being non-melanic (Table 4, Figure 4F). Wing loading increased the odds of being non-melanic (OR = 2.34) (Table 4). The average model showed good predictive performance, with an AUC of 0.789, indicating good discrimination between melanic and non-melanic individuals.

### 3.5. Sex-Specific Environmental Associations of Melanic Morphotypes

Canonical Correspondence Analysis (CCA) performed for females indicated a low association between the frequency of the melanic morphotype and the selected environmental variables. The total inertia (scaled Chi-square) was 0.781, of which 23.27% (0.182) was explained by the constrained variables, while 76.73% (0.600) remained unexplained. The first canonical axis accounted for all of the constrained variation, with an eigenvalue of 0.182, and the global test for model significance was non-significant (F = 1.213, *p* = 0.356). The permutation test for the first axis (CCA1) is also non-significant (F = 8.793, *p* = 0.341). The permutation test for individual environmental variables showed that none contributed significantly to explaining variation in color morphotype female frequency (Table 5).

**Figure 5:**
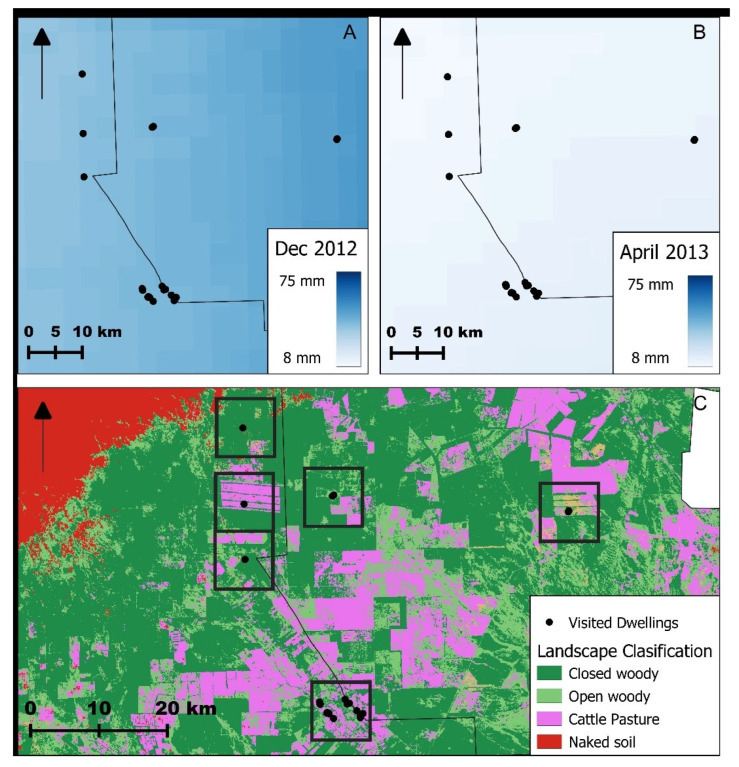
Variation in precipitation between the beginning of the warm season (December) (**A**), the end of the warm season (April) (**B**) and the vegetation cover for closed woody vegetation, open woody vegetation, cattle pastures and naked soils (**C**) in the rural study area of Córdoba, Argentina. The arrows on the top left indicates north.

**Figure 6:**
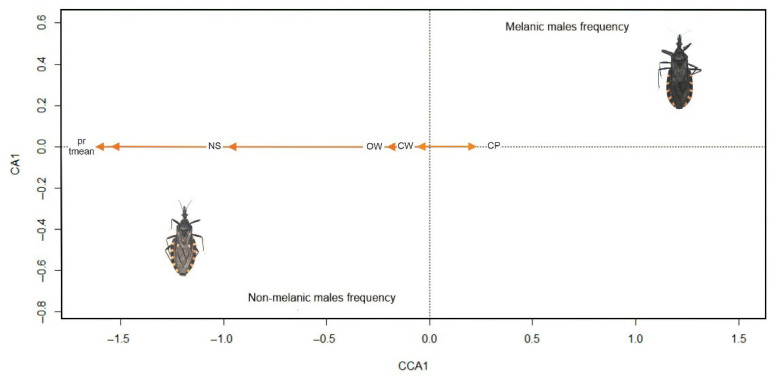
Canonical correspondence analysis (CCA) of chromatic morph frequencies and environmental predictors for males of *Triatoma infestans* collected in a well-defined rural area in Córdoba, Argentina. CCA 1 (first canonical axis) represents the variation in the male chromatic frequency that is explained by the environmental predictors. CA1 (first unconstrained (residual) axis) represents the variation not explained by the environmental variables. Environmental variables included: precipitation (pr), mean temperature (tmean), and the proportions of four vegetation cover categories—closed woody vegetation (CWV), open woody vegetation (OWV), cattle pasture (CP), and naked soil (NS).

The Canonical Correspondence Analysis (CCA) conducted for the frequency of melanic and non-melanic males showed a good association with the selected environmental variables (Table 5). The total inertia was 0.866, of which 45.5% (0.394) was explained by the constrained variables, while the remaining 54.5% (0.472) was not explained by the environmental variables included in the model. The first canonical axis (CCA1) accounted for all of the constrained variation, with an eigenvalue of 0.394. The global permutation test for model significance was statistically significant (F = 3.34, *p* = 0.011), suggesting that the model explained a significant proportion of the variation in color morphotype male frequency. Similarly, the test for the significance of individual axes showed that the first axis (CCA1) was statistically significant (*p* = 0.007). When testing the individual effect of each predictor, the percentage of cattle pastures (*p* = 0.004) and precipitation (*p* = 0.025) were found to be significant. Mean temperature showed a trend toward significance (*p* = 0.060). Other variables were not statistically significant (*p* > 0.1) (Table 5, Figure 5A–C). The relationship between chromatic morph frequencies of males and environmental variables showed that the frequency of melanic males has a positive score (0.554) while the frequency of non-melanic males has a negative score (−0.711) (Figure 6). Since the significant environmental variables—cattle pasture and precipitation—are positively associated with CCA1, higher values of these variables are associated with a higher frequency of melanic males, while lower values are associated with a higher frequency of non-melanic males.

## 4. Discussion

Our findings reveal clear seasonal and sex-specific patterns in the frequency and morphometric characteristics and flight estimators of chromatic morphotypes. The proportion of melanic individuals decreased progressively from the beginning to the end of the warm season, a trend particularly evident in males. Among the biological traits analyzed, flight-related features especially wing loading were strongly associated with chromatic morphotype, with higher wing loading linked to the non-melanic form. Morphological traits such as head and thorax measurements were important predictors of color morphotype, although their influence varied depending on the season and sex. In contrast, nutritional status did not appear to influence morphotype variation. Environmental variables, including rainfall and cattle pastures, may further modulate morphotype frequencies, especially in males. Overall, these results indicate that the expression and distribution of color morphotypes in this species is shaped by a combination of biological traits, seasonal dynamics, and environmental conditions. As hypothesized, our findings support the prediction that the frequency of melanic and non-melanic *T. infestans* forms varies between seasons and is associated with biological and environmental variables. Specifically, melanic individuals were more frequent during dry periods, and differed from non-melanic individuals in flight-related and morphometric traits.

### 4.1. Seasonal and Sex-Specific Variation in the Frequency of Chromatic Morphotypes

Seasonal and sex-specific variation in the frequency of chromatic morphotypes in *T. infestans* was observed, suggesting that sex-biased dispersal processes are modulated by seasonal dynamics. Specifically, we observed a significantly higher frequency of melanic males at the beginning of the warm season, followed by a marked decline towards its end, while melanic females maintained relatively stable frequencies throughout the same period. In many insect species, males are typically the dispersing sex, but *T. infestans* has shown variable patterns. Some studies report greater flight initiation in males across different ecotopes [7,20,62], while others suggest higher dispersal in females, based on greater flight muscle mass [72], more frequent flight initiation [31], and lower genetic and phenotypic structuring [73,74,75]. Furthermore, Nattero et al. [35] reported that females with developed flight muscles exhibit specific forewing traits that may enhance flight performance. Given that flight is the primary mechanism for dispersal and colonization in triatomines, often triggered by the search for food and mates, this temporal shift reinforces the notion that melanic males are more prone to seasonal dispersal, driven by reproductive strategies rather than survival-related factors [22,76]. In addition, dispersal behavior may also be modulated by environmental conditions, population density [22,77] and the attraction to artificial light, which promotes movement toward human dwellings [78,79]. The Arid Chaco ecoregion presents a markedly seasonal climate with hot and wet summers, and cold and dry winters that affect the life cycle and the flight dispersal process of triatomines. In the Argentine Arid Chaco, flight dispersal in *T. infestans* tends to peak during the summer, shows substantial variability across spring seasons [23], and remains at low levels by late autumn and winter, following contrasting seasonal patterns [20,80]. Flight initiation activity of *T. infestans* was predicted to peak in summer, when adults also peak in abundance and reach the lowest nutritional status (lowest weight-to-length ratio) [62]. Multiple factors may influence the seasonal flight dispersal pattern of *T. infestans*. In July (winter), the combination of low temperatures and a high nutritional status (high weight-to-length ratio) limits the insects’ dispersal ability [20]. In contrast, when temperatures are favorable for flight (above 22 °C), a higher proportion of adults and their lower nutritional status appear to be the main drivers of the late-summer dispersal peak. Active dispersal of triatomines between October and December is likely driven by both favorable climatic conditions and their life cycle [54,77]. Another key factor influencing dispersal seasonality, as proposed by Noireau and Dujardin [81], is the seasonal fluctuation in the availability of wild hosts such as rodents and marsupials. During the dry season, these hosts expand their spatial range, making blood meals less accessible. The food scarcity can lead to a critical nutritional state in triatomines that rely on these hosts, thereby promoting adult dispersal flights in search of new feeding sources [54].

### 4.2. Sex-Specific Environmental Associations of Melanic Morphotypes

The observed reversal in morphotype frequencies between sexes across the season likely reflects differing dispersal pressures and behaviors shaped by reproductive roles or resource needs, consistent with patterns observed in other triatomine species [82]. Our findings suggest that morphotype frequency is not a static population trait but is dynamically shaped by temporally varying pressures that differ between sexes. Climatic variables, particularly precipitation, emerged as influential factors, even within a relatively small rural area and a short collection interval of four months. Historical data show that average precipitation in December (53.77 mm) was considerably higher than in April (21.50 mm), which may have influenced triatomine behavior or morph distribution. These seasonal environmental changes are consistent with broader ecological theory. On a global scale, climate is a dominant driver of species distribution due to its variability across large geographic areas [83]. However, at regional and local scales, other ecological factors—such as land cover, surrounding habitat, and microclimatic conditions—become increasingly relevant [82,83,84,85,86]. Cattle pasture appears to influence the frequency of chromatic morphs, with a higher proportion of pasture associated with an increased frequency of the melanic morph. These results are consistent with previous field studies, indicating that human-driven management and alterations of rural and urban environments may impose selective pressures on Triatominae populations inhabiting domestic settings [52,87,88,89]. Land-use change represents a major driver of landscape transformation. Deforestation and habitat degradation alter landscape structure by reducing forest area and fragment size, increasing isolation, and amplifying edge effects [90]. These structural changes tend to negatively affect native species, including sylvatic triatomines [91], but may also create conditions favorable for domiciliary species like *T. infestans*. Although *T. infestans* is highly adapted to domestic environments, anthropogenic modifications—such as livestock pastures and agricultural zones—can shape population structure and promote colonization [52,87,89]. In fact, environmental changes can lead to increased triatomine densities even in less anthropized landscapes. For example, higher densities of *Triatoma garciabesi* Carcavallo, Cichero, Martínez, Prosen & Ronderos, 1967 have been reported in such areas [37], while other triatomine species, like *Rhodnius pallescens* Barber, 1932, have shown increased abundance in disturbed environments [92]. Similarly, in *Triatoma dimidiata* (Latreille, 1811), changes in land use and vegetation coverage at the municipal level in El Salvador were associated with variation in chromatic traits—melanic morphs were more common in areas with higher percentage of green space, natural vegetation, and agriculture [88]. Insects with melanic coloration are often better adapted to fluctuating environmental conditions. Melanism has been associated with enhanced thermoregulation, faster heat absorption, greater resistance to UV radiation, and altered tolerance to desiccation [93,94]. These traits may influence both the survival and spatial distribution of melanic morphs, particularly under conditions of seasonal environmental stress. Despite these potential advantages, fluctuating asymmetry analyses of melanic and non-melanic individuals collected at the beginning of the warm season in our study area suggest higher levels of developmental instability in melanic insects [50]. Taken together, our findings emphasize the importance of integrating temporal and sex-specific perspectives when interpreting the ecological and evolutionary mechanisms shaping chromatic morphotype variation in *T. infestans*. The consistent early-season abundance of melanic males, followed by their decline, suggests that environmental and behavioral drivers interact in complex ways that merit further investigation, particularly in light of the potential non-domestic origin of this morphotype.

### 4.3. Morphometric and Wing Loading Variations as Predictor of Chromatic Morphotypes

Our results consistently showed across seasons that both females and males melanic individuals had a greater distance between the humeri compared to non-melanic ones. For the female and males analyzed in this study, this measurement was significantly correlated with total body length in both chromatic morphotypes. Additionally, melanic individuals exhibited a greater total body length than non-melanic ones. This suggests that, in this case, the larger humeral width observed in melanic individuals may reflect overall body size rather than being a trait directly associated with flight performance, as proposed in previous studies [95]. For *T. infestans*, size reduction was reported for wings of domestic specimens collected from two sites in Cochabamba, Bolivia (Laguna Angostura and Jamach’uma) compared with sylvatic dark morphs collected 1 km away from these domestic foci [96]. In *Panstrongylus rufotuberculatus* (Champion, 1899) (Triatominae), a reduction in body size was observed in the transition from sylvatic to domestic habitats [97]. Regarding flight performance indicators, wing loading showed a significant association with morphotype differentiation, with non-melanic individuals displaying higher values for this parameter. Wing loading, defined as the ratio of body mass to wing area, is commonly used as an indicator of flight efficiency in various species. These metrics can be valuable for identifying potential differences in colonization success between species or sexes. Several studies have demonstrated that traits such as wing loading can influence flight performance and dispersal propensity, indicating a correlation between flight morphology and dispersal capability [31]. Taken together, these findings suggest that the non-melanic morphotype may possess a greater dispersal capacity compared to the melanic morphotype.

The colorimetric quantification technique proposed in this study has proven effective in previous research for detecting and quantifying color variation in *T. infestans* [50,98]. For example, Sánchez Casaccia et al. [98] applied this method to investigate the potential origin of reinfesting insects in 12 de Junio, an Indigenous community in Paraguay’s central Chaco. Their analysis revealed shifts in chromatic morphotype frequencies before and after insecticide spraying, with melanic individuals being more common in wild environments. These results suggest insect movement between habitats and highlight the potential role of wild areas as sources of (peri)domestic reinfestation. Similar findings have been reported in Argentina, Bolivia, and Paraguay [47,48,99], where melanic individuals—referred to as “dark morphs” due to their overall darker coloration and reduced, darker yellow connexivum markings—predominate in sylvatic populations. In rural Córdoba, Nattero et al. [50] also identified two distinct chromatic morphotypes, with melanic individuals exhibiting greater wing asymmetry. Melanic individuals collected from peridomestic environments in our study area share several of these features. However, it remains unclear whether they are genetically related to the dark morphs from wild populations. This represents one of the main limitations of our study, as no genetic analyses were conducted to confirm whether the melanic forms observed in peridomestic settings correspond to those described in sylvatic habitats. Clarifying this would allow for more definitive conclusions about the origin and potential role of these individuals in reinfestation dynamics. Furthermore, although we identified significant morphometric differences between chromatic morphotypes, a direct experimental assessment of flight performance across morphs remains lacking. Future experimental studies that examine the relationship between chromatic phenotype, flight capacity, and dispersal will be essential to better understand the role of melanic individuals in post-control recolonization. Although the collections analyzed in this study were conducted over a decade ago, chromatic dimorphism in *T. infestans* remains a recurrent phenomenon in (peri)domestic areas of the study region and elsewhere. In the department of Cruz del Eje, adult specimens collected in 2018 and 2025 continue to exhibit chromatic variation patterns similar to those described here. Additionally, we have observed chromatic dimorphism in adult *T. infestans* from a defined rural area in the province of Chaco, within the Argentine Gran Chaco (Nattero et al., unpublished data). All these records indicate that chromatic dimorphism is a persistent and geographically widespread trait in *T. infestans*. As such, studying this form of phenotypic variation remains highly relevant, and the findings presented here continue to offer valuable insights into the dispersal dynamics of this species.

## 5. Conclusions

Our study highlights the complex interplay between biological traits, seasonal environmental dynamics, and landscape factors in shaping chromatic morphotype variation in *T. infestans*. The consistent early-season predominance of melanic males and their subsequent decline, along with morphological and flight-related differences between morphotypes, suggests that dispersal behavior may be modulated by both sex-specific strategies and environmental cues. While melanic individuals were generally larger in body size, non-melanic morphs exhibited higher wing loading values, indicating a potentially greater dispersal capacity. Climatic variables, such as precipitation, and land-use patterns—including the presence of livestock pastures—also appear to influence morphotype distribution, particularly in males. Although our findings provide valuable insights into phenotypic variation and potential dispersal mechanisms, further genetic and experimental studies are needed to clarify the origin and ecological significance of the melanic morphotype. Understanding these dynamics is crucial for improving vector surveillance and control strategies, especially in regions where sylvatic and peridomestic populations may interact and contribute to reinfestation after insecticide spraying campaigns.

## Figures and Tables

**Table 1:** Details from the origin and the chromatic morphotype of the studied individuals of *Triatoma infestans* collected in a defined rural area of the Cruz del Eje and Ischilín Department of Córdoba province, Argentina.

Sampling Periods	Number of Adults Collected	Sexes	Melanic Morphotype	Non-Melanic Morphotype
Beginning of the warm season (December 2012)	105	50 females 55 males	15 females 28 males	35 females 27 males
End of the warm season (April 2013)	61	28 females 33 males	12 females 6 males	16 females 27 males

**Table 2:** Results of the conditional average model (based on multi-model inference) describing the relationship between morphometric variables and the probability of being non-melanic in females and males of *Triatoma infestans* collected at the beginning of the warm season from a well-defined rural area in the Cruz del Eje and Ischilín Departments, Córdoba province, Argentina.

Sex	Variable	Estimate (β)	SE	z-Value	*p*-Value	OR (Odds Ratio)	95% CI (OR)	*p*-Value (OR)
Female	Interocular distance	0.980	0.550	0.564	0.081	3.314	1.12–12.28	0.046
	Anterocular distance	−0.920	0.516	1.741	0.082	0.424	0.13–1.19	0.115
	Membranous portion area	−2.737	2.717	0.981	0.327	0.204	0.00–52.78	0.593
	Distance humeri	−0.910	0.601	1.478	0.140	0.563	0.14–2.07	0.389
Male	Interocular distance	1.107	0.527	2.050	0.040	2.754	0.98–9.08	0.068
	Forewing length	0.904	0.816	1.082	0.279	0.692	0.21–2.13	0.565
	Forewing width	0.506	0.702	0.703	0.482	0.722	0.22–2.13	0.565
	Membranous portion area	4.718	4.517	1.019	0.482	22.87	0.01–3.55	0.473
	Distance humeri	−1.287	0.550	2.286	0.022	0.251	0.07–0.71	0.016

**Table 3:** Results of the conditional average model (based on multi-model inference) describing the relationship between morphometric variables and the probability of being non-melanic in females and males of *Triatoma infestans* collected at the end of the warm season from a well-defined rural area in the Cruz del Eje and Ischilín Departments, Córdoba province, Argentina.

Sex	Variable	Estimate (β)	SE	z-Value	*p*-Value	OR (Odds Ratio)	95% CI (OR)	*p*-Value (OR)
Female	Interocular distance	−0.016	0.590	0.027	0.9788	1.039	0.32–3.87	0.949
	Anterocular distance	0.191	0.730	0.250	0.803	1.975	0.33–20.98	0.482
	Forewing length	−0.516	0.792	0.621	0.534	0.373	0.02–2.55	0.392
	Distance humeri	−2.171	0.909	2.276	0.023	0.082	0.00–0.43	0.033
Male	Interocular distance	2.504	1.463	1.651	0.099	22.688	1.62–21.48	0.082
	Anterocular distance	0.634	1.529	0.401	0.688	0.766	0.03–0.52	0.024
	Forewing length	1.872	1.188	1.510	0.131	7.498	0.64–26.27	0.183
	Distance humeri	−1.977	0.938	2.028	0.043	0.104	0.01–0.52	0.024

**Table 4:** Results of the conditional average model (based on multi-model inference) describing the relationship between flight estimator variables and the probability of being non-melanic in females and males of *Triatoma infestans* collected in two sampling periods (beginning and end of the warm seasons) from a well-defined rural area of the Cruz del Eje and Ischilín Departments of Córdoba province, Argentina.

Sex	Variable	Estimate (β)	Std. Error	z-Value	*p*-Value	OR (Odds Ratio)	95% CI (OR)	*p*-Value (OR)
Female	Wing loading	1.829	0.440	4.091	0.000	6.353	2.87–17.15	0.000
	Aspect ratio	0.086	0.300	0.281	0.779	1.512	0.56–4.12	0.402
	Sampling	0.173	0.630	0.271	0.787	2.343	0.31–19.04	0.406
Male	Wing loading	0.844	0.304	2.705	0.007	2.341	1.35–4.61	0.006
	Aspect ratio	−0.079	0.372	2.322	0.203	0.385	0.09–1.06	0.094
	Sampling	−0.242	1.207	0.198	0.198	0.785	0.04–7.01	0.841

**Table 5:** Significance tests for environmental variables derive from the Canonical Correspondence Analyses performed on the frequency of female and male chromatic forms during each sampling season for each house where *Triatoma infestans* were collected in the rural study area of Córdoba, Argentina. Environmental variables included: precipitation (pr), mean temperature (tmean), and the proportions of four vegetation cover categories—closed woody vegetation (CWV), open woody vegetation (OWV), cattle pasture (CP), and naked soil (NS).

	Variable	Chi-Square Value	F	*p*-Value		Variable	Chi-Square Value	F	*p*-Value
Female	pr	0.011	0.433	0.528	Male	pr	0.117	5.942	0.025
	tmean	0.014	0.564	0.489		tmean	0.078	3.955	0.060
	CWV	0.002	0.083	0.806		CWV	0.001	0.009	0.931
	OWV	0.074	2.964	0.102		OWV	0.005	0.264	0.590
	CP	0.066	2.621	0.118		CP	0.166	7.441	0.004
	NS	0.015	0.612	0.466		NS	0.028	1.427	0.267

## Data Availability

Data supporting the conclusions of this article are included in the article and its additional files. The preprocessed data are available from the corresponding author on reasonable request.

## Supplementary Materials

The following supporting information can be downloaded at: https://www.mdpi.com/article/10.3390/insects16111103/s1, Table S1: Top-ranking models (ΔAICc < 3) explaining variation in morphometric traits in females and males of *Triatoma infestans* collected at the beginning of the warm season from a well-defined rural area of the Cruz del Eje and Ischilín Department of Córdoba province, Argentina. Table S2: Top-ranking models (ΔAICc < 3) explaining variation in morphometric traits in females and males of *Triatoma infestans* collected at the end of the warm season from a well-defined rural area of the Cruz del Eje and Ischilín Department of Córdoba province, Argentina. Table S3: Top-ranking models (ΔAICc < 3) explaining variation in flight estimator variables in females and males of *Triatoma infestans* collected in two sampling periods (beginning and end of the warm seasons) from a well-defined rural area of the Cruz del Eje and Ischilín Department of Córdoba province, Argentina.

## Abbreviations

The following abbreviations are used in this manuscript:

W	Body weight
L	Total bod length
DH	Distance between humeri
AD	Anterocular distance
MID	Interocular distance
WL	Forewing length
MW	Maximum width
FA	Forewing area
MA	Membranous area
SA	Stiff area
WL	Wing loading
AR	Aspect ratio
