# Supplementary material for: Environmental and Dispersal-Related Drivers of Color Morph Distribution in Triatoma infestans (Klug, 1834) (Hemiptera, Reduviidae)
Source: Insects. 2025 Oct 29;16(11):1103. doi: 10.3390/insects16111103 (PMC12653105; doi:10.3390/insects16111103)
Supplement: Supplementary file 1 [file insects-16-01103-s001.zip › Additional file 1.pdf]

Additional file 1.

Table S1.

Sex	Model ID	Predictors included	AICc	ΔAICc	Weight
Female	Best model	anterocular distance, interocular distance	61.1	0.00	0.227
	1	anterocular distance, interocular distance, distance between humeri	61.9	0.75	0.156
	2	interocular distance, distance between humeri	61.9	0.80	0.152
	3	interocular distance	62.1	0.96	0.140
	4	membranous portion area, anterocular distance, interocular distance	62.4	1.24	0.122
Male	Best model	interocular distance	72.7	0.00	0.317
	1	interocular distance, distance between humeri	73.6	0.88	0.204
	2	interocular distance, distance between humeri, forewing length	74.7	1.08	0.184
	3	interocular distance, forewing length	75.0	2.00	0.116
	4	membranous portion area, interocular distance	75.6	2.27	0.102
