# Supplementary material for: Environmental and Dispersal-Related Drivers of Color Morph Distribution in Triatoma infestans (Klug, 1834) (Hemiptera, Reduviidae)
Source: Insects. 2025 Oct 29;16(11):1103. doi: 10.3390/insects16111103 (PMC12653105; doi:10.3390/insects16111103)
Supplement: Supplementary file 1 [file insects-16-01103-s001.zip › Additional file 2.pdf]

Additional file 2.

Table S2.

Sex	Model ID	Predictors included	AICc	ΔAICc	Weight
Female	Best model	distance between humeri	28.7	0.00	0.363
	1	membranous portion area, distance between humeri	30.5	1.80	0.147
	2	stiff portion area, membranous portion area, distance between humeri	30.5	1.82	0.146
	3	stiff portion area, membranous portion area, distance between humeri	30.7	1.98	0.135
	4	interocular distance, distance between humeri	31.2	2.45	0.106
Male	Best model	interocular distance, forewing length, distance between humeri	26.9	0.00	0.370
	1	stiff portion area, interocular distance, forewing length, distance between humeri	27.7	0.84	0.243
	2	interocular distance, distance between humeri	28.3	1.45	0.179
	3	interocular distance, anterocular distance, distance between humeri	29.2	2.32	0.116
	4	interocular distance, anterocular distance, distance between humeri, forewing length	29.7	2.76	0.093
