# Supplementary material for: Environmental and Dispersal-Related Drivers of Color Morph Distribution in Triatoma infestans (Klug, 1834) (Hemiptera, Reduviidae)
Source: Insects. 2025 Oct 29;16(11):1103. doi: 10.3390/insects16111103 (PMC12653105; doi:10.3390/insects16111103)
Supplement: Supplementary file 1 [file insects-16-01103-s001.zip › Additional file 3.pdf]

Additional file 3.

Table S3.

Sex	Model ID	Predictors included	AICc	ΔAICc	Weight
Female	Best model	wing loading	75.3	0.00	0.587
	1	aspect ratio, wing loading	77.4	2.08	0.207
	2	aspect ratio, wing loading, sampling	77.4	2.09	0.207
Male	Best model	wing loading	101.9	0.00	0.746
	1	aspect ratio, wing loading,	104.0	2.15	0.254
